# Why is this happening to me? – a comparison of illness representations between Iranian and German people with mental illness

**DOI:** 10.1186/s40359-018-0250-3

**Published:** 2018-07-20

**Authors:** Judith Reichardt, Amrollah Ebrahimi, Hamid Nasiri Dehsorkhi, Ricarda Mewes, Cornelia Weise, Hamid Afshar, Peyman Adibi, Said Moshref Dehkordy, Gholamreza Yeganeh, Hanna Reich, Winfried Rief

**Affiliations:** 1 Division of Clinical Psychology and Psychotherapy, Department of Psychology, Philipps University Marburg, Gutenbergstraße 18, 35032 Marburg, Germany; 2 Psychosomatic Research Center, Isfahan University of Medical Sciences, Isfahan, Iran; 3 Gastroenterology research center, Isfahan University of Medical Sciences, Isfahan, Iran; 4 Faculty of Psychology, University of Vienna, Vienna, Austria

**Keywords:** Illness representations, Causal beliefs, Mental disorders, Cross-cultural comparison

## Abstract

**Background:**

Due to an increase in migration and globalization, cross-cultural encounters in health care are also becoming more frequent. As psychotherapy is grounded in a cultural context and must be congruent with the patient’s cultural beliefs of his or her illness in order to be effective, the consideration of cross-cultural differences in illness representations becomes increasingly important. Especially research on illness representations concerning mental disorders is scarce.

**Methods:**

The aim of the current study was to compare illness representations between Iranian (*N* = 87) and German (*N* = 90) patient samples as well as subclinical samples (Iranian *N* = 264, German *N* = 102) using a multivariate analysis of covariance (MANCOVA). Illness representations were measured using the Illness Perception Questionnaire Revised (IPQ-R). Initially, a factor analysis was conducted in order to ensure comparability of the IPQ-R between the Iranian and the German sample.

**Results:**

The factor analysis already revealed differences in item compositions of the IPQ-R subscales indicating differences of the conception of illness representations between the samples. Further, the Iranian samples showed a significantly higher amount of supernatural causal beliefs and emotional representation of the illness than the German samples. Surprisingly, the Iranian patient sample showed the highest amount of illness coherence.

**Conclusion:**

The current paper contributes to a deeper understanding of cross-cultural differences in illness representations regarding mental disorders. Nevertheless, further research is needed to confirm current findings and to further elaborate on the relationships found.

## Background

Culture shapes every aspect of psychiatric patient care [1] as well as the individual’s values, beliefs and practices [2]. Accordingly, also illness representations develop differently in people with diverse cultural backgrounds. Since illness representations have an important influence on health-related behaviors and the actual treatment adherence [3, 4], their consideration for the psychotherapeutic treatment is essential. To provide effective mental health care, a high congruence between a treatment and the individual’s culturally grounded illness representations is desired [5]. The increasing migration flows as well as general globalization make it therefore necessary to investigate cultural differences in illness representations in order to optimize mental health care. Mental disorders have a high prevalence worldwide, are one of the main causes of disability, and result in high direct and indirect costs for the health care systems [6–8]. Culturally adequate and effective treatment is therefore highly needed. To provide such a treatment insights into possible cultural differences in illness representations are highly relevant.

Illness representations are defined as “frameworks or working models that patients construct to make sense of their symptoms and medical conditions” (Petrie & Weinman, 2012, p. 60). They evolve depending on the personal experiences of a certain illness and the individual’s context [9], the information provided by relevant others (such as physicians, friends or relatives) [10], and the individual’s cultural background (e.g. the structure of the country-specific health care system, cultural beliefs about health and illness or typical linguistic expressions of symptoms) [11].

According to Leventhal’s Self-Regulatory-Model, illness representations encompass cognitive and emotional components [11]. *Cognitive illness representations* include assumptions about causes, consequences and illness duration, as well as beliefs about successful treatment options, the perceived amount of personal control, perceived coherence and the presumed outcome. The *emotional component* of illness representation encompasses fears or worries concerning the illness. Both components influence which behavioral and emotional coping strategies people apply, how they report symptoms or seek help [11, 12]. Although the Self-Regulatory-Model originally refers to physical illnesses its applicability to mental disorders is supported by many studies examining the effects of illness representations on health-related outcomes in patients needing psychiatric or psychotherapeutic care [13–16].

For Germany, cross-cultural comparisons have focused on Turkey, since most immigrants in Germany are Turkish. Results show that Turkish people have a more negative perception of their mental illness than German people, believing more often that they cannot control their illness and that their illness is caused by supernatural forces [17, 18]. Findings regarding emotional representation of mental disorders are inconsistent: Whereas Franz and Salize [17] found evidence for more worries and anxiety in Turkish compared to German patients, Lujic [19] did not.

Cross-cultural comparisons of illness representations concerning *mental* disorders focusing on Iranian population groups are lacking so far. Some studies have, however, investigated specific subparts of illness representations in Iranian population groups. For example, Vahabi [20] showed that Iranian women commonly assume that their breast cancer is God’s providence or caused by supernatural forces, which might be associated with the high importance of spirituality and religion in the Iranian culture and everyday life. In a similar manner it is assumed that for Iranian women religion is essential for coping with depression or seeking help [21]. Furthermore, the important role of family is discussed as an influencing factor on illness representations. Hence, family conflicts are frequently reported as causes for symptoms in Iranian population groups [21–23]. With regard to control beliefs (i.e. a person’s beliefs about the extent to which the course and outcome of an illness are controlled by internal or external factors) results are inconsistent, too: Lipson and Hafizi [24] found that Iranian people considered their personal responsibility in the treatment to be low (low internal control), whereas Aflakseir and Mohammad-Abadi [25] found high internal control as well as high control by God in an Iranian sample. In studies investigating German participants, in contrast, people report high personal control and rarely any supernatural causes for their illnesses [17, 26].

In summary there seem to be differences in illness representations between members of different societies. However, there is only little research on cross-cultural comparisons of illness representations concerning mental disorders in Iranian population groups. To the best of our knowledge, there have been no cross-cultural comparisons on illness representations between Iran and Germany so far. As Iran and Germany clearly differ in regards to socio-cultural, political and health care related realities, and since these realities influence the development of illness representations [10], cross-cultural differences in illness representations between Iranian and German patients are very likely. Further, Iran differs from other oriental countries, as it is not Arabic. That is why illness representations might be different as well. Therefore, results of studies comparing illness representations between Muslim and non-Muslim societies [27] are presumably not applicable. This study aims to approach this research gap. As the Self-Regulatory-Model was developed in and for western cultures, its applicability to non-western cultures is not self-evident. In preparation for our analyses we are thus investigating if illness representations are comparable in the Iranian and the German sample.

The aim of the current study was to compare illness representations regarding mental disorders between Iranian and German patients. We hypothesize that there will be statistically significant differences in coherence, causes, personal control and emotional representation between the samples. As illness representations influence an individual’s health care utilization, we were not only interested in whether illness representations differ between patients with mental disorders but also in people with subclinical symptoms. Further, the inclusion of Iranian and German subclinical samples was considered helpful to strengthen the generalizability of the findings.

## Methods

### Design and procedure

In the current cross-sectional study, we administered a set of questionnaires assessing socio-demographic information, psychological and physiological symptoms as well as illness representations to a convenience sample in Iran and Germany. Data was collected via an online survey using the platform SoSci Survey [28] or via paper pencil. Participants in both countries filled out web based and paper pencil surveys. The study was approved by the local ethics committees of the University of Marburg (chair: Prof. Dr. Lothar Schmidt-Atzert), reference number 2014–08-k, and the Medical University of Isfahan, reference number IR.MUI.REC.1394.1.73.

### Participants

Participants were recruited concurrently in Iran and Germany. Both samples (Iranian and German) include people from the general population as well as inpatients in treatment in psychiatric and psychosomatic hospitals with a diagnosis of anxiety or mood disorder (depression). Patients with an additional diagnosis of schizophrenia, bipolar disorder or dementia were not included, neither were participants with a migration background. The minimum age of all participants was 18 years. Participants of the population samples were recruited via Internet platforms (e.g. social networks) as well as mailing lists of the universities involved. They received a hyperlink to the online survey, hosted on http://soscisurvey.de. In both countries, patients with anxiety and/or mood disorders were recruited via their attending physicians or psychotherapists. They filled in a paper-pencil version of the survey, as Internet access could not necessarily be provided. All participants gave written informed consent before filling in the survey. Participants needed on average 30 min to complete the survey.

**Fig. 1:**
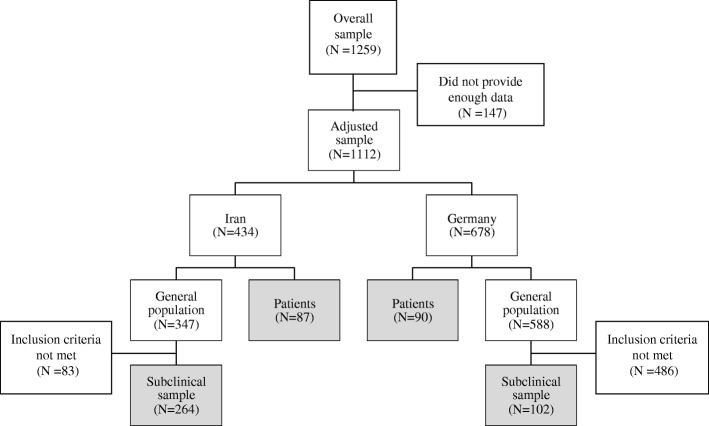
Flow chart of the sample composition

A total of 1259 German and Iranian people participated in our study (see Fig. 1). From the total sample, *N* = 147 people (German and Iranian) had to be excluded from the analyses because they did not provide complete data (more than 10% missing values), resulting in *N* = 1112 participants included in the analyses. Since the main goal of our study was to investigate illness representations in the two cultural samples, we needed to ensure that the answers of both population samples provide meaningful information concerning illness representations. Therefore, in the next step, we only included participants with at least mild impairment through symptoms of mental disorders assessed with the Patient Health Questionnaire (PHQ) and the Posttraumatic stress Diagnostic Scale (PDS). Participants were included in analyses if they met *at least one* of the following criteria: PHQ9 > 5 (Kroenke, Spitzer & Williams, 2001), GAD7 > 5 (Kroenke, Spitzer & Williams, 2010), PDS > 11(Foa, 1995), PHQ15 > 5 [29], DSM-IV criteria for panic disorder according to the PHQ-Panic, or criteria for binge eating disorder according to the PHQ-Eating. A total of 83 Iranian participants and 286 German participants of the general population had to be excluded because they did not meet the criteria of mild impairment. This resulted in the following sample sizes: Iranian subclinical sample *N* = 264, German subclinical sample *N* = 102, Iranian patients *N* = 87, German patients *N* = 90. For a detailed overview of the flow of participants, see Fig. 1.

### Measures

Participants filled in a German or a Farsi version of the survey. To receive a Farsi version of the respective questionnaires, members of the team of A.E. at the University of Isfahan, Iran, translated the English language questionnaires to Farsi. Two Iranian native speakers revised the resulting questionnaires.

#### Socio-demographics

The socio-demographic data included age, sex, level of education, and religiousness (“Without religious belief”, “Merely participating in religious duties”, “Believer in religion”).

### Classification / diagnoses of mental disorders

Patients’ diagnoses were received from the treating psychotherapists/physicians of the institution they were hospitalized in. As people of both population groups (German and Iranian subclinical samples) were mainly recruited via online survey they were not diagnosed. For the assessment of all further variables, questionnaires available and validated in German and English were used.

To assess symptoms of mental disorders, several subscales of the Patient Health Questionnaire (PHQ-D) [30] were used:

The *PHQ9* is a 9-Item scale to assess depressive symptoms according to the DSM-IV criteria on a four point Likert-scale. A higher score indicates more depressive symptoms. The scale shows good internal consistencies with *Cronbach’s α* = 0.88, in the current study internal consistencies of the samples ranged from acceptable to good (*α* = 0.76–0.87). The *GAD7* measures seven common anxiety symptoms (e.g. irritability or hypersensitivity) on a three point Likert-scale with higher scores indicating more or a higher intensity of anxiety symptoms. Internal consistencies range from *α* = 0.67 to 0.79 in our samples. The *PHQ-15* includes 15 of the most common somatoform symptoms that are rated on a three point Likert-scale, ranging from “not at all” to “affected a lot”. Due to very high missing rates especially in the Iranian samples, we excluded the item “Pain or problems during sexual intercourse”. In the current samples *Cronbach’s α* ranges from *α* = 0.61 to 0.79. The *PHQ-Panic* screens for the panic syndrome with 15 Items representing the DSM-IV criteria for panic disorder. It has a dichotomous response format (Yes/No). Accordingly, this subscale can be analyzed only categorically. The *PHQ-Eating* is a screening instrument for the binge eating disorder. It consists of eight items about eating and purging behavior with a Yes/No response format.

Moreover, the subscale *Somatization Symptom Count* of the Screening for Somatoform Symptoms (SOMS-7) [31] was included. The SOMS-7 screens for 53 physical symptoms on a five point Likert-scale with higher scores indicating more symptoms. We found internal consistencies ranging from α = 0.76 to 0.92 in our samples.

Furthermore, the subscale *Symptom Severity* of the *Posttraumatic Stress Diagnostic Scale* [32] was used to assess symptoms of the posttraumatic stress disorder. It consists of 17 items that are rated on a four point Likert-scale. Internal consistencies ranged from good to very good (*α* = 0.86–0.91).

In addition, illness representations concerning symptoms of mental disorders were assessed with the *Illness Perception Questionnaire Revised* [33]. A first section on general illness beliefs consisted of 38 Items that was rated on a five point Likert-scale (“Strongly disagree”, “Disagree”, “Neither agree nor Disagree”, “Agree”, “Strongly agree”). They can be summed up in seven subscales, which measure participants’ assumptions about course, consequences, controllability, and coherence (i.e. understanding) of their condition as well as related emotions. The subscales are Timeline acute/chronic (e.g. “My illness will last for a long time.”), Timeline cyclical (e.g. “My symptoms come and go in cycles.”), Consequences (e.g. “My illness has major consequences on my life.”), Emotional Representation (e.g. “Having this illness makes me feel anxious.”), Treatment Control (e.g. “My treatment will be effective in curing my illness.”), Personal Control (e.g. “I have the power to influence my illness.”), and Coherence (e.g. “I have a clear picture or understanding of my condition.”). People from the general population received a slightly adapted version of the IPQ replacing the words “my illness” with “my complaints” to take into account that people of the general population would not refer to their symptoms as “illness”. In contrast to the English original (Moss-Morris et al., 2002) from the German version [34] six items were removed due to poor factor loadings (<.50). In order to ensure comparability a Farsi translation of this version was used for data acquisition in Iran. In a second section, 18 causes that may be responsible for the illness can be rated on a five point Likert-scale. Due to the cross-cultural nature of this study we included five new items related to causes (“Evil eye/Maledictions”, “God’s will”, “Supernatural forces”, “My Gender” and “Being faint hearted”). These items had shown to be relevant for participants of collectivistic cultures in other studies (mostly Turkish) [26, 35].

Given the sufficient size (*N* > 90) of our sample, a factor analysis can be conducted to identify underlying dimensions. To ensure the comparability of the IPQ-R in our study, we conducted a principal component analysis with promax rotation in the German and the Iranian sample, respectively. As the results of a factor analysis including only the patient samples showed similar patterns as the factor analysis including all samples we decided to use the latter because of the significantly higher sample size. Only the subscales *Coherence*, *Emotional Representation* and *Personal Control* showed comparable item compositions and internal consistencies ranging from *α* = 0.82–0.95 in both samples, which is comparable to the original IPQ-R. In the other subscales (*Treatment Control, Consequences, Timeline acute/chronic and Timeline cyclical*) item compositions differed clearly between the samples, resulting in very poor internal consistencies (e.g. *α* = 0.54 for *Treatment Control*) and indicating a lack of comparability of those subscales. Based on these results we decided to limit the objectives of our study to three illness representation components (*Coherence*, *Emotional Representation* and *Personal Control)*, which showed highest comparability between our samples. Further factor analyses of the Cause-items showed that only one factor (consisting of the Items “Supernatural forces”, “God’s will” and “Evil eye/Maledictions”) was comparable between both groups. We named the factor “Supernatural Beliefs” and included it in the analysis.

### Statistical analysis

All analyses were performed using the Statistical Package for Social Sciences (SPSS; version 22, IBM, Chicago, Illinois, USA). To calculate differences between the groups, independent t-tests (age and mental stress), and *χ*^2^-analyses (educational level, religiousness, and sex) were conducted. In the subclinical samples, group differences with regard to the illness representation dimensions Coherence, Emotional Representation, Personal Control as well as Supernatural Beliefs were analyzed by using a multivariate analysis of covariance (MANCOVA) including group as a fixed factor. Demographic variables, which differed significantly between the groups, where included as covariates (sex and educational level, see Table 1). To examine the differences in the patient samples the same outcome variables were analyzed by using a multivariate analysis of covariance (MANCOVA) including group as a fixed factor. Age and educational level were included as covariates, because the groups differed significantly on these variables (see Table 1).

**Table 1:** Characteristics of the patient sample and the subclinical sample in Iran and Germany

	Subclinical sample			Patient sample		
	Iran	Germany	Statistical comparison	Iran	Germany	Statistical comparison
*N*	264	102		87	90	
Age (*M, SD* years)	29.6 (12.0)	28.0 (9.2)	*t*(222.5) = 1.38	34.2 (10.2)	41.0 (13.1)	*t*(169.6) = 3.92***
Sex (% female)	62.2	79.4	*χ*^*2*^ (2) = 17.13***	69.2	60.4	*χ*^*2*^ (1) = 1.54
Educational level (%)			*χ*^*2*^ (5) = 29.36***			*χ*^*2*^ (5) = 58.72***
Primary school	1.2	–		11.1	1.1	
Secondary school	1.2	–		6.7	26.7	
Diploma	28.8	52.9		23.3	54.4	
Associate degree	6.8	10.8		10.0	10.0	
Bachelor’s degree	34.0	11.8		35.6	2.2	
Master’s degree	28.0	24.5		13.3	5.6	
Religiousness (%)			*χ*^*2*^ (2) = 55.32***			*χ*^*2*^ (2) = 35.4***
Believer in religion	43.0	18.2		48.8	10.0	
Merely doing duties	40.8	27.3		39.3	53.3	
Without religious belief	16.2	54.5		11.9	36.7	
Diagnosis (%)						
Mood	–	–	–	64.8	77.8	
Anxiety	–	–	–	35.2	6.1	
Mixed	–	–	–	–	16.1	
Hospitalization (%)	–	–	–	31.7	53.5	*χ*^*2*^ (1) = 8.3**
Outpatient treatment (%)	–	–	–	72.1	67.4	*χ*^*2*^ (1) = 0.45
Mental stress (M, SD)						
PHQ9^a^	8.1 (5.3)	6.5 (4.0)	*t*(238.4) = 3.20**	14.0 (6.5)	7.6 (4.9)	*t*(167.2) = − 7.38***
GAD7^b^	5.2 (4.4)	4.1 (2.0)	*t*(358.6) = 8.45***	10.6 (5.8)	3.5 (2.3)	*t*(115.6) = −
PHQ15^c^	8.6 (4.7)	4.1 (2.0)		11.7 (5.1)	4.8 (3.3)	10.85***
SOMS7^d^	5.5 (6.1)	1.9 (2.4)	*t*(372.1) = 13.20***t	10.5 (8.6)	5.3 (5.8)	*t*(154.7) = 10.80***
			(372.4) = 8.19***			*t*(156.5) = 4.73***

*Note. N* = Sample size, *M* = Mean, *SD* = Standard deviation, *t* = *t* value, *χ*^*2*^ = Chi-square value, ^a^ Depression score of the PHQ, ^b^ Anxiety score of the PHQ, ^c^ Score for somatoform symptoms of the PHQ, ^d^ Score for somatization symptom count of the SOMS7 ^*^ *p* < 0.05, ^**^ *p* < 0.01, ^***^ *p* < 0.001

Due to the risk of *α*-error accumulation, the Bonferroni corrected significance value for the univariate statistics of the MANCOVAs was set to *p* < 0.0125. The *p* value for other analyses was set to *p* < 0.05. *Cohen’s d* is reported as measure for effect size, whereby *d* = 0.20 is referring to a small effect, *d* = 0.50 to a moderate effect and *d* = 0.80 to a large effect [36].

## Results

### Sample characteristics

Sample characteristics are shown in Table 1. The Iranian and German samples differed from each other in the subclinical as well as the patient sample: The patient samples had a higher educational level and a lower age in the Iranian sample than in the German one. Concerning the subclinical sample there were more females in the German sample than in the Iranian one and a higher educational level in the Iranian than the German sample. In the subclinical as well as the patient samples, Iranians reported a higher level of mental stress as well as a higher level of religiousness than Germans. Concerning the patient samples, more Germans than Iranians were hospitalized at least once, whereas there were no differences in whether they were or had been in outpatient treatment at least once.

### Comparison of illness representations in the patient samples

Concerning the comparison of the Iranian and German patient samples, the MANCOVA showed a large significant main group effect for the observed dimensions of illness representations, *T* = 2,01, *F* (4, 170) = 85,50, *p* < 0.001, *d* = 2.85. Subsequent univariate ANOVAs revealed a significant group effect for differences between the patient samples on the IPQ subscales Coherence, Emotional Representation, and Supernatural Beliefs. See Table 2 for details. Iranian patients scored higher than German patients on all three dimensions. High effect sizes with *d* = 0.92 for Coherence, *d* = 1.6 for Emotional Representation and *d* = 2.53 for Supernatural Beliefs underline these findings. In addition, no group differences for the subscale Personal Control were found in the patient samples.

**Table 2:** Comparisons of illness representations between the Iranian and German subclinical samples and between the Iranian and German patient samples

	Subclinical sample^a^			Patient sample^b^				
	Iran	Germany	*F*(1, 362)	*d*	Iran	Germany	*F*(1, 362)	*d*
IPQ-R scales (*M, SD*)								
Coherence	3.43 (0.83)	3.76 (0.94)	10.22^**^	0.38	4.12 (0.62)	3.40 (0.91)	36.16^***^	0.92
Personal control	3.21 (0.73)	3.21 (0.85)	0.04 n.s.	0.00	3.42 (0.84)	3.32 (0.74)	0.04 n.s.	0.00
Emotional representation	3.83 (1.00)	3.29 (0.97)	22.15^***^	0.54	4.71 (0.56)	3.62 (0.78)	98.27^***^	1.6
Causes (*M, SD*)								
Supernatural beliefs	3.12 (1.02)	1.26 (0.55)	299.42^***^	2.0	3.52 (0.88)	1.53 (0.68)	264.97^***^	2.53

*Note.* ^a^ including covariates sex and educational level, ^b^ including covariates age and educational level, *M* = Mean, *SD* = standard deviation, *F* = *F* value, *df =* degrees of freedom, ^*^ *p* < 0.0125, ^**^ *p* < 0.0025, ^***^ *p* < 0.00025, *d* = Cohen’s d

### Further analyses of the patient samples

To further examine the relationship between the IPQ subscales (Coherence, Emotional Representation, and Supernatural Beliefs) and certain sample characteristics, bivariate correlations were conducted (see Table 3). In the German patient sample, Emotional Representation was significantly associated with anxiety symptoms (GAD7 sum score), depressive symptoms (PHQ9 sum score) as well as somatoform symptoms (SOMS7, PHQ15 sum score). In addition religiousness was positively related to IPQ-Supernatural Beliefs. Concerning the intercorrelations of the illness representation dimensions only Coherence and Emotional Representation were correlated.

**Table 3:** Correlation matrix of IPQ subscales and sample characteristics of the German patient sample

	Age^a^	Sex^b^	Educational level^b^	Religiousness^b^	PHQ9^a^	GAD7^a^	PHQ15^a^	SOMS7^a^	Coherence^a^	Emotional representation^a^
Age^a^	–									
Sex^b^	0.15	–								
Educational level^b^	−0.08	0.00	–							
Religiousness^b^	0.30**	0.17	− 0.14	–						
PHQ9^a^	− 0.01	0.08	0.10	0.02	–					
GAD7^a^	0.07	0.04	−0.01	0.08	0.72**	–				
PHQ15^a^	0.19	0.22*	−0.04	0.14	0.66**	0.70**	–			
SOMS7^a^	0.16	0.30**	−0.16	0.11	0.48**	0.45**	0.75**	–		
Coherence^a^	−0.02	0.14	0.03	−0.05	−0.19	− 0.20	−0.11	− 0.14	–	
Emotional representation^a^	−0.18	0.001	−0.08	0.06	0.38**	0.47**	0.38**	0.30**	−0.21*	–
Supernatural beliefs^a^	0.13	−0.01	−0.07	0.19*	0.02	−0.09	0.10	0.11	−0.06	0.01

*Note*. *N* = 90, ^a^ Pearson’s product-moment correlation, ^b^ Kendall’s tau, * *p* < 0.05, *** *p* < 0.001

In the Iranian patient sample we found significant correlations between illness representation dimensions and certain sample characteristics as well (see Table 4): the IPQ subscale Emotional Representation was significantly associated with anxiety symptoms (GAD7 sum score), depressive symptoms (PHQ9 sum score), and somatoform symptoms (SOMS7, PHQ15 sum score). Furthermore, IPQ-Supernatural Beliefs was associated with depressive symptoms (PHQ9 sum score), and somatoform symptoms (SOMS7, PHQ15 sums score). Regarding intercorrelations between the illness representation dimensions, Coherence as well as Supernatural Beliefs were positively associated with Emotional Representation.

**Table 4:** Correlation matrix of IPQ subscales and sample characteristics of the Iranian patient sample

	Age^a^	Sex^b^	Educational level^b^	Religiousness^b^	PHQ9^a^	GAD7^a^	PHQ15^a^	SOMS7^a^	Coherence^a^	Emotional representation^a^
Age^a^	–									
Sex^b^	−0.03	–								
Educational level^b^	−0.35**	−0.01	–							
Religiousness^b^	0.16	0.08	−0.19	–						
PHQ9^a^	−0.08	−0.06	0.04	0.03	–					
GAD7^a^	0.07	0.03	−0.07	0.11	0.53**	–				
PHQ15^a^	0.14	0.22*	−0.13	0.01	0.40**	0.31**	–			
SOMS7^a^	0.28**	0.04	−0.21**	0.08	0.33**	0.35**	0.71**	–		
Coherence^a^	0.17	0.10	−0.15	0.10	0.004	0.02	−0.01	0.12	–	
Emotional representation^a^	0.10	0.13	−0.07	0.03	0.27**	0.24*	0.24*	0.25*	0.53**	–
Supernatural beliefs^a^	−0.01	0.12	−0.11	0.10	0.21*	−0.06	0.22*	0.31**	0.14	0.30**

*Note. N* = 87, ^a^ Pearson’s product-moment correlation, ^b^ Kendall’s tau ^*^ *p* < 0.05, ^**^ *p* < 0.01

### Comparison of illness representations in the subclinical samples

Using Hotellings’s trace statistic, there was a large significant main group effect on the observed dimensions of illness representations, *T* = 0.866, *F* (4, 359) = 77.86, *p* < 0.001, *d* = 1.85. Subsequent univariate ANOVAs revealed significant group effects for the IPQ subscales Coherence, Emotional Representation, and Supernatural Beliefs. For further details of the analysis, see Table 2. German participants scored higher on Coherence, whereas Iranian participants scored higher on Emotional Representation and Supernatural Beliefs. Effect sizes for the differences in the subscales Coherence (*d* = 0.38) and Emotional Representation (*d* = 0.54) were small to moderate whereas the effect for Supernatural Beliefs was large (*d* = 2.0). For the subscale Personal Control, no significant group effect was detected (*d* = 0.0), indicating highly similar estimates for personal options to influence the symptoms.

## Discussion

The aim of this study was to investigate cross-cultural differences in illness representations concerning mental disorders in Iran and Germany. As expected, several differences were found, especially in the attribution of causes and the emotional representation of a mental disorder as well as the perceived coherence.

### Differences in illness representations

The Iranian samples showed higher scores in Supernatural Beliefs and Emotional Representation than the German samples. There were, however, no differences in Personal Control between the samples. The finding that Iranians assume more supernatural causes for their mental disorders than Germans is in line with the findings of Vahabi [20] that for Iranian women God’s providence and supernatural forces do play an important role in explaining their breast cancer. A commonly used explanation is the strong general influence of religion and spirituality in this culture. In contrast, spirituality and religion play a decreasing role in the everyday life of Germans [37], which could explain the low relevance of supernatural causal beliefs in the German samples. The differences in emotional representation of the illness are likely to be caused by an overall significantly higher level of mental stress in the Iranian samples, considering the high correlation between mental stress indicators (PHQ9, GAD7) and emotional representation.

The finding that our Iranian and German samples (patient and subclinical) did not differ in their perceived personal control is rather surprising when taking into account that previous studies did find higher levels of personal control in western compared to non-western cultures [26]. One reason for our finding could be that the Iranian samples had a relatively high level of education implying a higher income and thus more options to influence their lives, along with a higher feeling of control overall [38, 39]. The fact that there were no differences in personal control neither between the patient nor the subclinical samples indicates the generalizability of this finding.

With regard to coherence, differences between our Iranian and German samples were surprising: Whereas mean values of both, the Iranian and German subclinical samples as well as the German patient sample were quite similar and comparable to those in other studies [14], the mean value of coherence in the Iranian patient sample was considerably higher. This stands in contrast to other findings which suggest that a higher amount of coherence goes along with less mental stress [40]. This interesting new finding should be further investigated, focusing for instance on cultural characteristics in the understanding of coherence. It is possible that Coherence means something else in Iran than it does in Germany. Or, there actually is another culturally specific relationship between Coherence and mental health in Iran, as opposed to Germany. Further, it is possible that questionnaire related data is more influenced by religious and social expectations in Iran than in Germany, which could have lead to a stronger cultural influence on the response behavior of Iranian participants. The fact that not all of the IPQ-R subscales could be found in the Iranian sample further indicates that there are different conceptions of illness representations and that the IPQ-R needs cultural adaption.

### Shortcomings of the current study

Several limitations of the study should be noted. First, its cross-sectional design limits cause-and-effect interpretation (e.g. the cultural background as a cause for differences in illness representations). Second, all measures were in self-report format, thus shared-method variance may be related to some of the observed associations. Third, we did no backward translation of the survey’s Farsi version, which could have compromised equivalence of measurements. Moreover, it could not be determined with certainty that each measure is culturally sensitive. Culturally sensitive means that members of different linguistic or cultural groups understand the items and test results in the same way, which is a central criterion for the comparability of test results [41]. After all, Mewes et al. [42] found that the PHQ9 and PHQ15 are invariant of measurement for Germans and migrants in Germany. To maximize the comparability between the samples we included only those scales of the IPQ-R in our analyses, which showed a similar item composition and a minimum of good reliability in all samples.

### Advances and implications of the current study

The results of the current study have several theoretical and practical implications. First, to the best of our knowledge, this is the first study which has investigated differences in illness representations between Iranian and German samples. Moreover, we compared not only patient but also subclinical samples from Iran and Germany, which indicates the generalizability of our results. As a further strength of the current study, it contributes to a deeper understanding of illness representations regarding *mental* disorders, a field in which little research has been conducted. Especially our findings that only some of the IPQ-R subscales showed comparable item compositions between the samples (Coherence, Personal control, Emotional representation) indicate cultural differences in the conception of illness representations. The current study has the characteristics of a pilot study, because it is the first to compare illness representations regarding mental disorders between Iran and Germany. Further the IPQ-R is not sufficiently validated for Iranian samples. That is why further research is needed to confirm current findings and to further elaborate on the relationships found.

## Conclusions

Illness representations regarding mental illnesses seem to vary between the Iranian and German culture. These findings have implications for the psychotherapeutic and psychiatric care: As psychotherapy is grounded in a cultural context and must be congruent with the patient’s cultural beliefs of his or her illness to be effective [5], an adaption of psychotherapeutic treatment approaches is necessary when applied in a cultural context different to the one where the treatment was developed. Further, illness representations should be assessed individually to provide a meaningful contribution to therapy. Additionally, further research is needed to investigate if and how illness representations change for example in the context of migration and the permanent exposure to a different cultural context. This is relevant, especially with regard to the increasing number of refugees and their need for psychotherapeutic care. Even if the patient and clinician share the same ethnic or linguistic background, culture impacts health care through other influences of identity, for example gender, age or sexual orientation [43]. As diversity in societies increases individual results are needed to adapt and individualize psychotherapy and thus improve psychotherapeutic treatment.

## Abbreviations

ANOVA: Analysis of Variance
GAD7: Anxiety Section of the Patient Health Questionnaire
IPQ-R: Illness Perception Questionnaire Revised
MANCOVA: Multivariate Analysis of Covariance
PDS: Posttraumatic stress Diagnostic Scale
PHQ: Patient Health Questionnaire
PHQ15: Somatoform Section of the Patient Health Questionnaire
PHQ9: Depression Section of the Patient Health Questionnaire
SOMS-7: Screening for Somatoform Symptoms
